# Complete Genome Sequence of a Novel Strain of Cyanobacterium, *Anabaena* sp. 4-3

**DOI:** 10.1128/genomeA.00842-16

**Published:** 2016-08-18

**Authors:** Sarah Pfeffer, Steven Sowa, R. Malcolm Brown

**Affiliations:** University of Texas at Austin, Molecular Biosciences, College of Natural Sciences, Austin, Texas, USA

## Abstract

We report the complete nucleotide sequence of *Anabaena* sp. 4-3, an efficient producer of sucrose. It was isolated from salt flats near the University of Texas Marine Science Institute in Port Aransas, Texas. The genome may provide insight into the utilization of cyanobacteria as a source for biofuels.

## GENOME ANNOUNCEMENT

Cyanobacteria (formerly blue-green algae) are an ancient group of diverse photosynthetic microorganisms which encompass a wide range of morphologies (1, 2). The extraordinary adaptive ability of these organisms has allowed them to survive in a wide range of aquatic and terrestrial ecosystems. They can be found in soil environments, fresh and salt water systems, and even extreme habitats such as hot springs and soda lakes (1, 2). Under saline conditions, cyanobacteria have developed mechanisms to maintain osmotic balance with the external environment through the accumulation of solutes internally (3). The genus, *Anabaena*, in particular has used this ability to survive in intertidal habitats where fluctuations between saline and freshwater environments occur frequently. The cell adapts by accumulating sucrose internally when exposed to saltwater and secreting it into the external milieu when exposed to freshwater (3). Sucrose osmolytes accumulated by *Anabaena* and other cyanobacteria are of particular interest as a potential raw material for bioethanol. Investigations into the genome of *Anabaena* sp. 4-3 may provide vital information for the understanding of this important evolved adaptation (4–6).

We have sequenced the genome of *Anabaena* sp. 4-3. This particular strain was isolated from salt flats located near the University of Texas Marine Science Institute in Port Aransas, Texas (N27°48.064′ W97°05.950′). The DNA was extracted and subjected to sequencing using the Illumina HiSeq 2500 sequencer (University of Texas at Austin, ICMB Core Facility). The reads were assembled into contigs using Velvet 1/2/02 (7) and downloaded into Geneious 8.1.2 (8), and open reading frames (ORFs) were predicted using Glimmer (9).

The DNA sequence of *Anabaena* sp. 4-3 was resolved, and bioinformatics analysis revealed that it is approximately 5.61 Mbp in size with a G+C content of 41.4%. A total of 5,411 ORFs were identified in the genome and the complete annotation of the full genome is in progress.

### Accession number(s).

This whole-genome shotgun project has been deposited in DDBJ/ENA/GenBank under the accession no. LUHJ00000000. The version described in this paper is the first version LUHJ01000000.
